# Relationship Between Blood Concentrations of Hepcidin and Anemia Severity, Mycobacterial Burden, and Mortality Among Patients With HIV-Associated Tuberculosis

**DOI:** 10.1093/infdis/jiv364

**Published:** 2015-07-01

**Authors:** Andrew D. Kerkhoff, Graeme Meintjes, Rosie Burton, Monica Vogt, Robin Wood, Stephen D. Lawn

**Affiliations:** 1 Department of Medicine, University of California San Francisco School of Medicine; 2 Department of Global Health, Academic Medical Center, Amsterdam Institute for Global Health and Development, University of Amsterdam, The Netherlands; 3 The Desmond Tutu HIV Centre, Institute of Infectious Disease and Molecular Medicine; 4 Department of Medicine, Faculty of Health Sciences; 5 Clinical Infectious Diseases Research Initiative, Institute of Infectious Disease and Molecular Medicine, University of Cape Town; 6 Department of Medicine, Khayelitsha District Hospital, Cape Town, South Africa; 7 Department of Medicine, Imperial College; 8 Department of Clinical Research, Faculty of Infectious and Tropical Diseases, London School of Hygiene and Tropical Medicine, United Kingdom

**Keywords:** HIV, AIDS, tuberculosis, anemia, hepcidin, antimicrobial, Africa

## Abstract

**Background:**

Anemia is very common in patients with human immunodeficiency virus (HIV)–associated tuberculosis, and hepcidin may be key in mediating this. We explored the relationship between blood hepcidin concentrations and anemia severity, mycobacterial burden and mortality in patients with HIV-associated tuberculosis.

**Methods:**

Consecutive unselected HIV-infected adults in South Africa were systematically investigated for tuberculosis. Three groups were studied: 116 hospitalized inpatients with HIV infection and tuberculosis (hereafter, “hospitalized patients”), 58 ambulatory outpatients with HIV infection and newly diagnosed tuberculosis (hereafter, “ambulatory patients with tuberculosis”), and 58 ambulatory outpatients with HIV infection and without tuberculosis (hereafter, “ambulatory patients without tuberculosis”). Blood hepcidin concentrations were determined for all patients. Vital status at 3 months was determined, and independent predictors of mortality were identified.

**Results:**

Median hepcidin concentrations were 38.8 ng/mL among hospitalized patients, 19.1 ng/mL among ambulatory patients with tuberculosis, and 5.9 ng/mL among ambulatory patients without tuberculosis (*P* < .001). In both groups with HIV-associated tuberculosis, hepcidin concentrations were strongly associated with greater anemia severity. Additionally, strong, graded associations were observed between hepcidin and composite indices of mycobacterial burden and dissemination. Patients dying within 3 months had significantly higher hepcidin concentrations, which independently predicted mortality.

**Conclusions:**

High hepcidin concentrations were strongly associated with disseminated disease, anemia, and poor prognosis in patients with HIV-associated tuberculosis. Hepcidin may be a mechanistically important mediator underlying the high prevalence of severe anemia in these patients.

**(See the editorial commentary by Armitage and Moran on pages 3–5.)**

Tuberculosis remains the leading cause of death among people living with human immunodeficiency virus (HIV) globally and accounts for approximately 300 000 AIDS-related deaths each year in sub-Saharan Africa [1]. Among HIV-infected patients, low hemoglobin levels are strongly predictive for tuberculosis [2–4], and anemia is one of the most common complications of both disease processes [5–7]. In patients with HIV-associated tuberculosis, anemia is strongly associated with morbidity and risk of death [8–11]. Thus, there is a need for a better understanding of the mechanisms underpinning anemia in such patients so that effective interventions may be designed and implemented. These mechanisms are likely to be multifactorial, although increasing evidence suggests that a majority of patients with HIV-associated tuberculosis have anemia of chronic disease (ACD) with or without an additional etiology [12, 13].

Hepcidin is an acute-phase reactant peptide that is the central regulator of iron homeostasis, and its expression is modulated by several factors, including body iron status and hypoxia [14]. Additionally, infections and inflammation may stimulate hepcidin expression by hepatocytes, a process that is mediated via proinflammatory cytokines, notably interleukin 6 (IL-6), and signaling through the STAT-3 pathway [15, 16]. Hepcidin drives the process of ACD by causing iron to be diverted from the circulation and sequestered within cells of the reticuloendothelial system and by limiting duodenal absorption of iron. Thus, as a consequence of inflammation, hepcidin restricts the availability of iron for incorporation into erythroid progenitor cells [17].

In addition to its central role in iron regulation, hepcidin has antimicrobial properties and appears to have an important role in the innate immune response against *Mycobacterium tuberculosis* [15, 18–20]. While a small number of clinical studies have demonstrated an association between elevated hepcidin concentrations and tuberculosis [12, 21, 22], much of our knowledge regarding the role of hepcidin in tuberculosis has been derived from studies conducted in vitro or in animal models. Thus, we sought to further extend this work to well-characterized patient populations with newly diagnosed HIV-associated tuberculosis.

We hypothesized that elevated hepcidin concentrations would be associated with the degree of disease dissemination, as well as with a greater prevalence and severity of anemia in patients. Additionally, since anemia and disseminated tuberculosis are both associated with increased mortality risk, we further examined whether hepcidin had prognostic value. In the present study, we investigated these questions in well-characterized cohorts of patients with HIV-associated tuberculosis and differing disease severity (ambulatory vs hospitalized patients) in Cape Town, South Africa.

## METHODS

This study comprised hospitalized inpatients with HIV infection and newly diagnosed tuberculosis (hereafter, “hospitalized patients”), ambulatory antiretroviral therapy (ART)–naive outpatients with HIV infection and newly diagnosed tuberculosis (hereafter, “ambulatory patients with tuberculosis”), and ambulatory ART-naive outpatients with HIV infection and without tuberculosis (hereafter, “ambulatory patients without tuberculosis”). All patients were unselected and consecutively recruited as part of two previously reported studies of tuberculosis diagnostic analyses [23, 24], and most resided in the township communities of Cape Town, where there is a large tuberculosis burden [25, 26]. These studies were approved by the research ethics committees of the University of Cape Town and the London School of Hygiene and Tropical Medicine. All patients provided written informed consent.

Hospitalized patients were drawn from among HIV-infected patients from the same township communities who were admitted to medical wards at G. F. Jooste Hospital, ≥18 years old, without a tuberculosis diagnosis prior to admission, and systematically and thoroughly screened for tuberculosis regardless of clinical presentation [24]. These patients were eligible for inclusion in the present study if they were found to have a new microbiologically confirmed diagnosis of tuberculosis, had a frozen plasma sample available for laboratory measurements, and had not had a blood transfusion within the 120-day period (the approximate life span of red blood cells) prior to study entry.

We also included ambulatory patients with and without tuberculosis, who would be expected to have less severe disease than inpatients [23, 27]. Ambulatory patients were recruited from among ART-naive, HIV-infected patients aged ≥18 years who presented for ART initiation at the Hannan Crusaid HIV Centre and had a frozen serum sample available for laboratory measurements. All were systematically screened and tested microbiologically for tuberculosis regardless of clinical presentation, to carefully define the group with and the group without active tuberculosis. An equal number of each were included, such that the total number of ambulatory patients equaled the number of hospitalized patients studied. Ambulatory patients with and those without tuberculosis were matched to hospitalized patients (1:1 ratio of ambulatory to hospitalized patients) on the basis of age (±1 year), sex, and CD4^+^ T-cell count (±25 cells/µL) but not hemoglobin level.

### Procedures and Data Collection

Demographic and clinical details were obtained from all participants. Two sputum samples (spot and induced samples) were obtained whenever possible. Urine samples were also obtained from all patients and stored at −20°C. For hospitalized patients, a 5.0-mL venous blood sample was inoculated into BACTEC Myco/F Lytic culture vials (Becton Dickinson, Franklin Lakes, New Jersey) for incubation. An additional venous blood sample was collected from all patients for additional laboratory measurements, and plasma or serum specimens were stored at −80°C.

Full blood counts, creatinine levels, plasma viral loads, and blood CD4^+^ T-cell counts at hospital admission or at the first clinic visit prior to ART initiation were measured by the National Health Laboratory Service (NHLS) in Cape Town. The results of all mycobacterial investigations and all laboratory investigations were extracted from the NHLS computerized data system. Clinical outcomes of hospitalized patients 90 days after study entry were determined from patient case notes, the medical register, and electronic health record databases, whereas clinical outcomes of ambulatory patients were determined at follow-up visits during routine care as described previously [28]; patients were classified as alive, dead, or lost-to-follow-up.

### Laboratory Procedures

All clinical specimens for mycobacteriology were processed at centralized accredited NHLS laboratories according to standardized protocols and quality assurance procedures as previously described [23, 27]. In brief, sputum samples were decontaminated, pelleted by centrifugation, examined for acid-fast bacilli by using fluorescence microscopy, and tested for *M. tuberculosis* by using the Xpert MTB/RIF assay (Cepheid, Sunnyvale, California) and by liquid culture with mycobacterial growth indicator tubes (MGIT; Becton Dickinson, Sparks, Maryland). Any culture isolate positive for acid-fast bacilli was speciated to identify *M. tuberculosis* complex. Urine samples from all patients were tested using the Xpert MTB/RIF assay.

Plasma (from hospitalized patients) and serum samples (from ambulatory patients) were tested to determine concentrations of hepcidin and C-reactive protein (CRP). The concentrations of hepcidin were measured using the commercially available hepcidin 25 enzyme-linked immunosorbent assay (ELISA; DRG Instruments, Marburg, Germany); this assay uses a monoclonal antibody and has no known cross-reactivity with *M. tuberculosis* antigens. CRP concentrations were measured using the human CRP Quantikine ELISA (R&D Systems, Minneapolis, Minnesota). Normal ranges for hepcidin concentrations were defined as 0.6–23.3 ng/mL for males and 0.5–23.2 ng/mL for females [29]. All assays were performed in strict accordance with the manufacturers' instructions.

### Definitions and Statistical Analysis

A new tuberculosis diagnosis among ambulatory patients with tuberculosis was defined as the detection *M. tuberculosis* in at least one sputum sample by culture; among hospitalized patients, a new diagnosis was defined as the detection of *M. tuberculosis* in any clinical sample (sputum or nonrespiratory sample) by culture or the Xpert MTB/RIF assay. Patients were defined as tuberculosis negative if all samples tested negative for *M. tuberculosis.* Any patients with nontuberculous mycobacteria isolated from their samples were excluded. Pulmonary tuberculosis was defined as a positive sputum culture or Xpert assay result, and extrapulmonary tuberculosis was defined as a positive culture or Xpert assay result for any nonrespiratory sample; any patient with both pulmonary and extrapulmonary tuberculosis was categorized as having extrapulmonary tuberculosis. Disseminated tuberculosis was defined as the detection of *M. tuberculosis* by culture or the Xpert assay in specimens from 2 different anatomical compartments (eg, sputum, urine, and blood).

Anemia severity was classified according to World Health Organization criteria [30] as follows: no anemia, hemoglobin level of ≥13.0 g/dL for men and ≥12.0 g/dL for females; mild anemia, 11.0–12.9 g/dL for men and 11.0–11.9 g/dL for women; moderate anemia, 8.0–10.9 g/dL for both sexes; and severe anemia, <8.0 g/dL for both sexes. We used blood creatinine levels to calculate an estimated glomerular filtration rate (eGFR), using the Modification of Diet in Renal Disease Study equation [31].

Either χ^2^ or Fisher exact tests, for comparison of proportions, and Wilcoxon rank sum or Kruskal–Wallis tests, for comparison of medians, were used as appropriate. Spearman rank correlation coefficients were used to test the relationship between hepcidin concentrations and CRP concentrations. Box and whisker plots were used to examine the relationship between hepcidin concentrations and anemia severity, indices of mycobacterial burden, and mortality. The corresponding medians were compared using Wilcoxon rank sum or Kruskal–Wallis tests. Cox proportional hazards regression analyses were used to determine independent predictors of mortality at 3 months. Patients with unknown outcomes at 3 months were censored on the date they were last seen alive in a healthcare setting. Two multivariable models were constructed. The first included covariates in the univariable model meeting a predetermined *P* value cutoff of ≤.1, as well as a priori risk factors (age, CD4^+^ T-cell count, HIV load, hemoglobin level, eGFR, and CRP level). The second model was a backward stepwise elimination model that removed variables not meeting the predefined *P* value exit criterion of .1 in the multivariable model. Multivariable fractional polynomial modeling was used to explore whether there was evidence for a nonlinear relationship between hepcidin concentrations and mortality. All statistical tests were 2 sided with an α level of 0.05.

## RESULTS

Of 139 hospitalized patients with newly diagnosed tuberculosis who were potentially eligible, 8 did not have a plasma sample available for additional laboratory measurements, and 15 had received a blood transfusion within the previous 120 days. Thus, 116 hospitalized patients (83.5%) were included. An equal number of ambulatory ART-naive patients (n = 116) were also included; of these, 58 had confirmed tuberculosis, and 58 did not have tuberculosis.

Baseline characteristics for each patient population are shown in Table 1. In view of patient matching, hospitalized patients had similar ages, sex distribution, and CD4^+^ T-cell counts as ambulatory patients. Hemoglobin levels were lowest among hospitalized patients and highest among ambulatory patients without tuberculosis (Table 1). CRP concentrations were highest among hospitalized patients and nearly 2-fold higher than those among ambulatory patients with tuberculosis (*P* = .028), while CRP concentrations were much higher among ambulatory patients with tuberculosis, compared with ambulatory patients without tuberculosis (7.5-fold difference; *P* < .001). Among hospitalized patients, those who were ART naive tended to have lower CD4^+^ T-cell counts, higher viral loads, and higher CRP concentrations than those who were ART experienced. However, hemoglobin levels and hepcidin concentrations did not differ by ART status (data not shown).

**Table 1.** Baseline Characteristics, by Study Population

Characteristic	Hospitalized Patients (n = 116)	Ambulatory Patients With Tuberculosis (n = 58)	Ambulatory Patients Without Tuberculosis (n = 58)
Age, y^a^	34.3 (27.0–40.9)	33.9 (28.2–42.4)	33.8 (28.5–40.1)
Female sex^a^	77 (66.4)	38 (65.5)	36 (62.1)
ART status			
Naive	56 (48.3)	100	100
Current	45 (38.8)	…	…
Interrupted	15 (12.9)	…	…
Previously treated for tuberculosis	44 (37.9)	14 (24.1)	20 (34.5)
HIV parameter			
CD4^+^ T-cell count, cells/µL^a,b^	87 (33–186)	109 (42–203)	73 (32–195)
HIV load, log_10_ copies/mL^c^	5.0 (3.2–5.7)	4.9 (4.6–5.4)	4.8 (4.2–5.2)
Hematological parameter			
Hemoglobin level, g/dL	8.8 (7.5–10.8)	10.8 (8.7–11.8)	11.8 (10.5–12.8)
MCV, fL^d^	85 (79–89)	84 (79–89)	89 (83–92)
MCHC, g/dL^d^	32.9 (32.0–33.7)	33.6 (32.7–33.9)	33.4 (32.6–34.1)
WBC count, ×10^9^ cells	7.2 (5.0–9.9)	5.8 (4.7–8.1)	4.8 (3.7–5.8)
Platelet count, ×10^9^ cells	247 (177–334)	307 (210–419)	283 (212–347)
Hepcidin level, ng/mL	38.8 (15.7–88.8)	19.1 (9.4–53.0)	5.9 (2.8–11.1)
Kidney function			
Creatinine level, µmol/L^e^	62 (52–86)	65 (58–82)	68 (56–77)
eGFR, mL/min/1.73 m^2e^	132 (95–168)	132 (91–154)	126 (109–155)
Inflammatory parameter			
CRP level, mg/L^d,f^	109.0 (65.0–183.9)	60.2 (20.3–174.2)	8.0 (3.4–30.8)

Data are median (interquartile range) or no. (%) of patients. “Hospitalized patients” were hospitalized inpatients with HIV infection and tuberculosis, “ambulatory patients with tuberculosis” were ambulatory ART-naive outpatients with HIV infection and tuberculosis, and “ambulatory patients without tuberculosis” were ART-native outpatients with HIV infection but without tuberculosis.

Abbreviations: ART, antiretroviral therapy; CRP, C-reactive protein; eGFR, estimated glomerular filtration rate; HIV, human immunodeficiency virus; MCHC, mean corpuscular hemoglobin concentration; MCV, mean corpuscular volume; WBC, white blood cell.

^a^ The Kruskal–Wallis test for difference in median values of matched variables across the 3 patient population yielded the following: *P* = .949 for age, *P* = .852 for sex, and *P* = .883 for CD4 count.

^b^ One result was missing for a hospitalized patient.

^c^ Four results were missing for hospitalized patients.

^d^ Two results were missing for hospitalized patients.

^e^ One result was missing for an ambulatory patient with HIV infection and without tuberculosis.

^f^ One result was missing for an ambulatory patient with HIV infection and tuberculosis.

### Hepcidin Concentrations

The median hepcidin concentration was higher among ambulatory patients with tuberculosis (19.1 ng/mL), compared with that among ambulatory patients without tuberculosis (5.9 ng/mL; *P* < .001; Table 1). However, hospitalized patients had a median hepcidin concentration of 38.8 ng/mL, which was 2.0-fold higher than that of ambulatory patients with tuberculosis (*P* < .001). Thus, hepcidin concentrations were higher among patients with active tuberculosis, particularly those who had more-severe clinical illness requiring admission. Hepcidin concentrations were highly correlated with CRP concentrations among hospitalized patients (rho = 0.630; *P* < .001), ambulatory patients with tuberculosis (rho = 0.621; *P* < .001), and ambulatory patients without tuberculosis (rho = 0.315; *P* = .016).

### Relationship Between Hepcidin Concentrations and Anemia Severity

**Figure 1.**
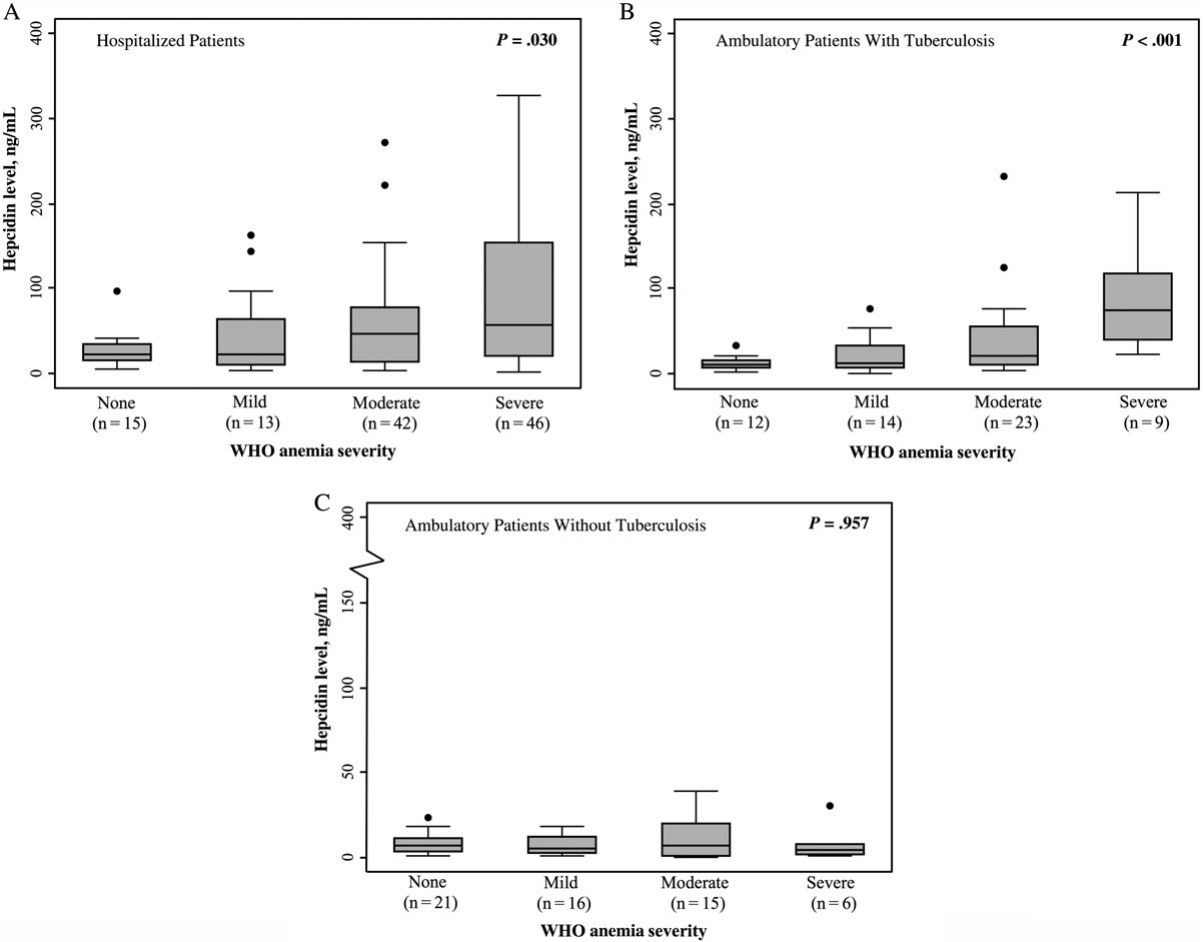
Box and whisker plots of hepcidin concentrations in patients with human immunodeficiency virus (HIV)–associated tuberculosis in different clinical settings, stratified by World Health Organization (WHO) anemia severity classification. *A*, Hospitalized patients with HIV infection and confirmed tuberculosis (n = 116). *B*, Ambulatory patients with HIV infection and confirmed tuberculosis (n = 58). *C*, Ambulatory patients with HIV infection but without pulmonary tuberculosis (n = 58). Bars, boxes, whiskers, and dots indicate medians, 25th and 75th centiles, ranges, and outliers, respectively. *P* values were calculated by Kruskal–Wallis tests for comparison of medians.

The relationship between hepcidin concentrations and anemia severity is shown in Figure 1. Among hospitalized patients (Figure 1*A*) and ambulatory patients with tuberculosis (Figure 1*B*), direct, graded positive associations between hepcidin concentrations and anemia severity were observed. The highest median hepcidin concentration was observed among those with severe anemia, and this was 2.5-fold and 4.0-fold higher than the concentrations among hospitalized and ambulatory patients with tuberculosis but without anemia, respectively. Hepcidin concentrations were not associated with anemia severity in ambulatory patients without tuberculosis (Figure 1*C*).

### Relationship Between Hepcidin Concentrations and Mycobacterial Burden

Among hospitalized patients, hepcidin concentrations varied substantially according to the results of tuberculosis diagnostic assays done on samples obtained from a range of different anatomical sites (Table 2). Median hepcidin concentrations were highest in those with positive urine Xpert assay and blood culture results, and these concentrations were significantly higher than those among patients with negative urine Xpert assay and blood culture results, respectively. In contrast, although numbers were small, patients with positive cerebrospinal fluid cultures and positive pleural fluid cultures had substantially lower median hepcidin concentrations compared to those with positive blood cultures and were within normal population ranges. Very similar relationships between hepcidin concentrations and the results of tuberculosis diagnostic assays were observed among ambulatory patients with tuberculosis (Supplementary Table 1).

**Table 2.** Relationship Between Hepcidin Concentrations and Tuberculosis Assay Results at Different Anatomical Sites Among Hospitalized Inpatients With Human Immunodeficiency Virus Infection and Tuberculosis

Specimen, Test, Result	Hepcidin Level, ng/mL, Median (IQR)	Fold-Difference (*P*)
Respiratory samples		
Sputum smear microscopy (n = 61)		
Positive (n = 34)	50.8 (18.4–96.8)	2.2 (.201)
Negative (n = 27)	23.1 (11.0–69.7)	
Sputum Xpert assay (n = 60)		
Positive (n = 47)	52.2 (23.8–96.8)	2.5 (.121)
Negative (n = 13)	20.6 (12.4–53.8)	
Sputum culture (n = 60)		
Positive (n = 45)	52.2 (20.6–96.5)	2.3 (.074)
Negative (n = 15)	23.0 (9.9–69.7)	
Nonrespiratory samples		
Blood culture (n = 113)		
Positive (n = 31)	61.0 (23.8–145.8)	1.8 (.029)
Negative (n = 82)	34.8 (12.4–77.3)	
CSF culture (n = 17)		
Positive (n = 7)	11.1 (6.0–32.8)	0.2 (.064)
Negative (n = 10)	47.2 (14.7–167.2)	
Pleural fluid (n = 13)		
Positive (n = 12)	16.0 (11.7–31.6)	8.4 (.109)
Negative (n = 1)	1.9	
Urine Xpert assay (n = 114)		
Positive (n = 74)	53.2 (21.9–142.9)	2.7 (.002)
Negative (n = 40)	19.9 (9.8–51.0)	

Abbreviations: CSF, cerebrospinal fluid; IQR, interquartile range.

**Figure 2.**
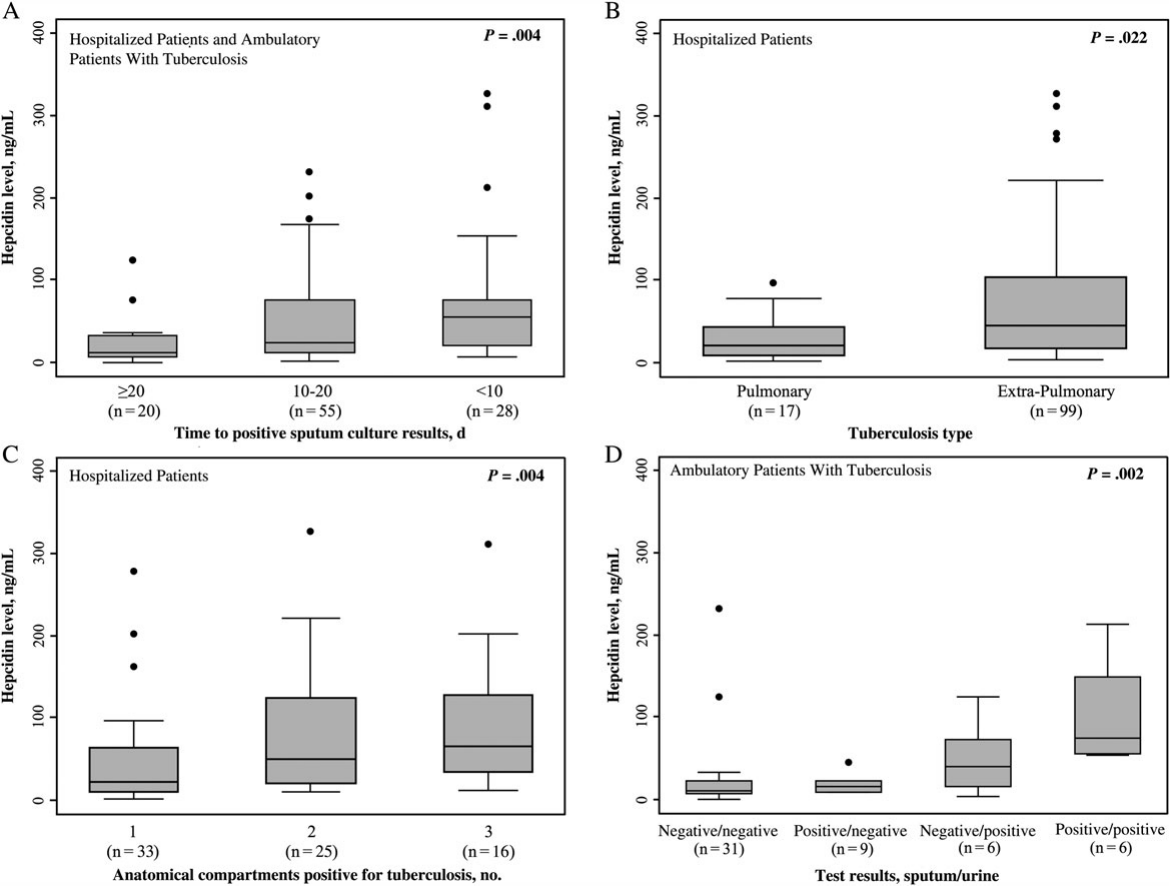
Box and whisker plots of hepcidin concentrations in patients with human immunodeficiency virus (HIV)–associated tuberculosis in different clinical settings, stratified by indices of mycobacterial burden. *A*, Time to sputum culture positivity among hospitalized patients and ambulatory patients with HIV infection and confirmed tuberculosis (n = 103). *B*, Tuberculosis classification among hospitalized patients with HIV infection and confirmed tuberculosis (n = 116). *C*, Number of unique anatomical compartments with confirmed tuberculosis among hospitalized patients with complete sputum, blood, and urine results (n = 74). *D*, Composite index of mycobacterial load combining sputum smear microscopy and urine Xpert assay results among ambulatory patients with HIV infection and confirmed tuberculosis (n = 52). Bars, boxes, whiskers, and dots indicate medians, 25th and 75th centiles, ranges, and outliers, respectively. *P* values were calculated by either Wilcoxon rank sum tests or Kruskal–Wallis tests for comparison of medians.

Next we investigated the relationship between hepcidin concentrations and additional indices of mycobacterial burden and disease dissemination (Figure 2). Among hospitalized patients and ambulatory patients with tuberculosis who had a positive sputum culture result, there was a strong association between times to sputum positivity and hepcidin concentrations (Figure 2*A*). Hepcidin concentrations among hospitalized patients were much higher in patients with extrapulmonary tuberculosis than in those with pulmonary tuberculosis (Figure 2*B*). Additionally, hospitalized patients who had tuberculosis detected in samples obtained from ≥2 anatomical compartments (such as sputum, urine, or blood), suggesting disseminated tuberculosis, had much higher hepcidin concentrations than those with more-localized disease confined to a single anatomical site (Figure 2*C*). When an index of mycobacterial burden was constructed among ambulatory patients with tuberculosis by combining sputum smear microscopy and urine Xpert assay results, hepcidin concentrations directly correlated with more-disseminated disease and were highest in patients with both a positive sputum smear and urine Xpert assay result (Figure 2*D*).

### Relationship Between Hepcidin Concentrations and Mortality

**Figure 3.**
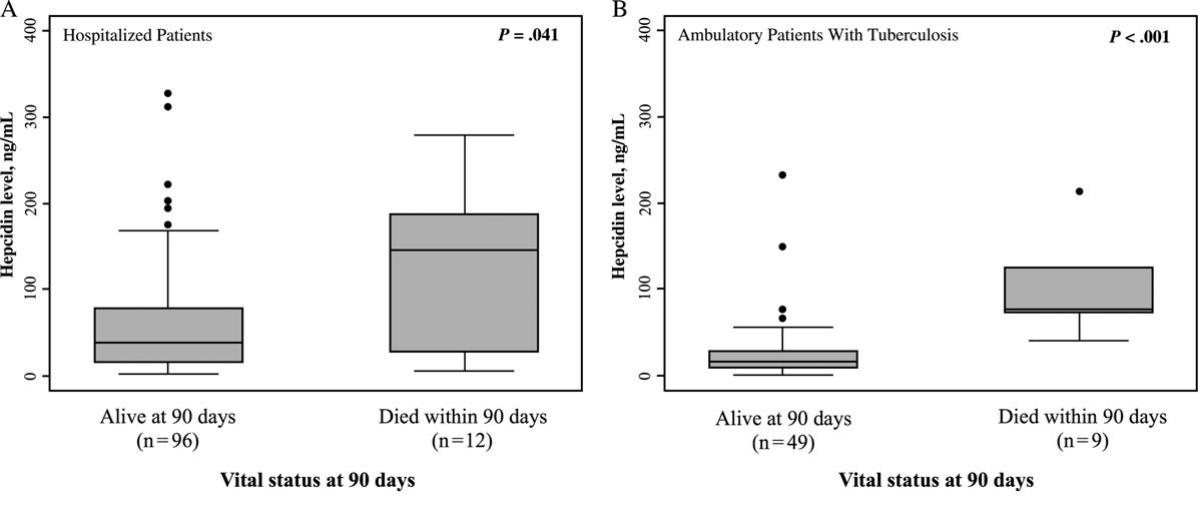
Box and whisker plots of hepcidin concentrations in patients with human immunodeficiency virus (HIV)–associated tuberculosis in different clinical settings, stratified by vital status at 90 days. *A*, Hospitalized patients with HIV infection and confirmed tuberculosis (n = 116). *B*, Ambulatory patients with HIV infection and confirmed tuberculosis (n = 58). Bars, boxes, whiskers, and dots indicate medians, 25th and 75th centiles, ranges, and outliers, respectively. *P* values were calculated by Wilcoxon rank sum tests for comparison of medians.

Twelve hospitalized patients (10.3%) and 9 ambulatory patients with tuberculosis (15.5%) died within 90 days of study entry; no ambulatory patients without tuberculosis died during this follow-up interval. Eight hospitalized patients (6.9%) and one ambulatory patient with tuberculosis were lost to follow-up within 90 days. The median hepcidin concentration among hospitalized patients who died was 3.9-fold higher than that among those who remained alive (144.6 ng/mL vs 37.3 ng/mL; *P* < .041; Figure 3*A*). Similarly, the median hepcidin concentration among ambulatory patients with tuberculosis who died was 5.0-fold higher than that among those who remained alive (75.8 ng/mL vs 15.2 ng/mL; *P* < .001; Figure 3*B*).

Multivariable Cox regression analyses were undertaken on data from all 174 patients with tuberculosis in the combined hospitalized and ambulatory groups to identify whether elevated hepcidin concentrations independently predicted mortality (Table 3). In both multivariable models, increasing hepcidin concentrations were independently associated with greater mortality risk.

**Table 3.** Cox Model for Predictors of Mortality Among 174 Hospitalized and Ambulatory Patients With Newly Diagnosed Human Immunodeficiency Virus (HIV)–Associated Tuberculosis

Predictor	Unadjusted HR (95% CI)	*P* Value	Adjusted HR 1 (95% CI)^a^	*P*	Adjusted HR 2 (95% CI)^b,c,d^	*P* Value
Age in y, per unit increase^e^	0.99 (.95–1.04)	.759	0.99 (.94–1.05)	.846	…	
Sex						
Male	1	.976	…		…	
Female	1.01 (.41–2.51)		…		…	
ART status						
Current	1	.714	…		…	
Naive	1.20 (.44–3.31)		…		…	
Interrupted	0.58 (.07–4.98)		…		…	
Patient type^e^						
Hospitalized inpatient	1	.332	1	.020	1	.022
Ambulatory outpatient	1.54 (.65–3.67)		4.23 (1.30–13.81)		3.27 (1.20–8.93)	
Disseminated tuberculosis^e^						
No	1	<.001	1	.426	…	
Yes	3.30 (1.19–9.17)		1.65 (.46–1.01)		…	
HIV parameters						
CD4^+^ T-cell count in cells/µL, per 10-unit decrease^e^	1.11 (1.03–1.20)	.003	1.12 (1.30–13.81)	.006	1.13 (1.04–1.23)	<.001
HIV load in copies/mL, per unit increase^e^	1.09 (.79–1.51)	.583	0.69 (.47–1.03)	.074	0.69 (.47–1.02)	.073
Hematological parameter						
Hemoglobin level in g/dL, per unit decrease^e^	1.27 (1.03–1.55)	.017	0.93 (.69–1.25)	.630	…	
Hepcidin level in ng/mL, per 10-unit increase^e^	1.09 (1.05–1.14)	<.001	1.08 (1.01–1.16)	.029	1.11 (1.05–1.17)	<.001
Kidney function^e^						
eGFR in mL/min/1.73 m^2^, per 10-unit decrease^e^	1.05 (.96–1.15)	.309	1.01 (.91–1.11)	.877	…	
Inflammatory marker						
CRP level in mg/L, per 10-unit increase^e^	1.05 (1.02–1.09)	.010	1.02 (.97–1.07)	.478	…	

Abbreviations: ART, antiretroviral therapy; CI, confidence interval; CRP, C-reactive protein; eGFR, estimated glomerular filtration rate; HR, hazard ratio.

^a^ There were 155 patients who had complete results and were included in the final model.

^b^ There was no evidence of collinearity among any of the variables included in the final model.

^c^ There was no evidence of interaction between hepcidin and any variables included in the final model. No evidence was found to suggest a nonlinear relationship between hepcidin concentrations and mortality.

^d^ There were 173 patients who had complete results and were included in the final stepwise backward elimination model.

^e^ A priori variables.

## DISCUSSION

Emerging evidence suggests a key role for the iron-regulator hepcidin in the innate immune response to *M. tuberculosis* infection [18]. In the present study, we demonstrated that hepcidin concentrations were strongly associated with mycobacterial burden and disseminated tuberculosis. Hepcidin concentrations were also positively associated with greater anemia severity among patients with tuberculosis. Finally, increased hepcidin concentrations independently predicted poorer short-term survival among hospitalized patients.

In patients with tuberculosis, higher hepcidin concentrations were strongly associated with more-severe anemia, and the highest hepcidin concentrations in both hospitalized and ambulatory patients were among those with severe anemia (hemoglobin level, <8.0 g/dL). Since hepcidin has a well-described, central role in ACD [17], in which its expression is upregulated predominantly by IL-6 in response to infections such as tuberculosis [14, 15], these results provide further evidence to suggest that ACD is the predominant mechanism underlying anemia in patients with HIV-associated tuberculosis [12, 13]. Furthermore, the observation that tuberculosis treatment with or without ART results in normalization of hepcidin concentrations and is associated with resolution of anemia in a majority of patients provides further indirect evidence for ACD as the predominant mechanism of anemia in patients with tuberculosis [12, 13, 32].

Of note, hepcidin concentrations demonstrated no association with anemia severity among HIV-infected ambulatory patients without tuberculosis. Anemia is common among HIV-infected patients [5], and increased iron stores are associated with HIV replication [33, 34]. Reduction of intracellular iron inhibits HIV transcription, and thus hepcidin may increase transcription by inhibiting ferroportin expression and not allowing iron efflux from cells [35]. Hepcidin also directly increases HIV replication in HIV-infected macrophages and T cells [35]. It is unclear why hepcidin was not associated with anemia severity among ambulatory patients without tuberculosis in our study, as it is commonly thought that ACD is the most important mechanism underlying anemia in HIV-infected patients. In HIV-infected patients without tuberculosis, a key mediator of anemia may, rather than hepcidin, be the direct infection of hematopoietic progenitor cells with HIV or the indirect effects of proinflammatory cytokines on hematopoietic progenitor cells causing dysregulated erythropoiesis [36]. Additionally, the relatively small number of patients studied may have limited the ability to detect a true association between hepcidin concentrations and anemia severity. The role of hepcidin during the natural history of HIV disease without tuberculosis or opportunistic infections and its downstream consequences (ie, anemia) merits future study.

Hepcidin concentrations were significantly higher among HIV-infected patients with tuberculosis, compared to among those without tuberculosis, suggesting that hepcidin concentrations are related to tuberculosis and are predictive of incident tuberculosis [21, 37]. However, hepcidin also appears to be related to the severity of tuberculosis, as indicated by the observation of strong, graded associations between hepcidin concentrations and several different indices of disease dissemination/mycobacterial load. Several mycobacterial components directly stimulate hepcidin release from lung macrophages, alveolar epithelial cells, and dendritic cells [19, 20]. Additionally, it has previously been demonstrated that hepcidin causes growth inhibition and structural damage to *M. tuberculosis* bacilli [19]. This evidence collectively suggests that hepcidin may be an important facet of the innate host response to *M. tuberculosis,* especially among HIV-infected patients with highly disseminated disease.

Of further interest was the observation of greatly differing hepcidin concentrations among the sites of tuberculosis involvement. Hepcidin concentrations were highest among those with renal tuberculosis (urine Xpert assay positive) or mycobacteremia. Relatively low concentrations were observed among those with confirmed but isolated tuberculosis in cerebrospinal fluid or pleural fluid, possibly indicating relatively compartmentalized disease and disease with lower mycobacterial load [38]. Previous studies have observed strong tuberculosis-specific immune responses induced at the local site of infection that are not reflected in systemic circulation [39].

Hepcidin concentrations were strongly prognostic in both patient populations with HIV-associated tuberculosis. Among West African HIV-infected patients, higher hepcidin concentrations were associated with mortality only in crude but not adjusted analyses [40]. Elevated hepcidin concentrations may have prognostic value for other diseases, as well, including chronic kidney disease and non-Hodgkin lymphoma [41, 42]. Anemia is associated with increased mortality risk among tuberculosis patients with and without HIV infection [10, 11] and disseminated tuberculosis disease, especially in HIV-infected patients, also confers a high mortality risk [11, 43]. Our results demonstrate that hepcidin expression is directly related to the degree of tuberculosis dissemination and, as an undesired consequence of hepcidin's role in the host response to disseminated mycobacterial disease, may result in ACD. Therefore, both hepcidin (upstream) and anemia (downstream) have prognostic value in HIV-associated tuberculosis, as they are both related to greater tuberculosis severity. It is worth noting, however, that in this study, anemia was not an independent predictor of mortality after adjustment for hepcidin and other possible confounding variables, suggesting that hepcidin is more predictive than hemoglobin levels of mortality in this patient population.

A major strength of this study was that relationships with hepcidin concentrations were explored in three well-characterized patient groups. This allowed us to compare findings in patients from different clinical settings (inpatient and outpatient), which were largely in agreement and improves the generalizability of our findings. Furthermore, because of a very thorough tuberculosis screening strategy in the parent studies, we were able to explore the relationship between hepcidin and confirmed tuberculosis in several different anatomical compartments. A limitation is that no single measure of mycobacterial burden exists, although microscopy, Xpert assay, and culture each have well-described lower limits of detection that are strongly correlated with known concentrations of mycobacteria [44]. As hepcidin and CRP levels were measured in plasma samples for hospitalized patients, this may have resulted in a slight underestimation of concentrations, compared with values for ambulatory patients, for whom serum specimens were measured (<10% difference) [45]; however, the majority of analyses and the key findings were derived from within-group analyses and could therefore not have not been affected by this. Given the observational design of this study, we were only able to report associations with hepcidin concentrations, but our results are consistent with in vitro studies and those conducted using animal models.

Hepcidin concentrations were strongly and positively associated with mycobacterial burden, suggesting that hepcidin may have a role in the host response to disseminated tuberculosis in HIV-infected patients. As hepcidin was also strongly correlated with more severe anemia, this implies that anemia may be a common and unfavorable consequence of this response. Therefore, hepcidin may be a mechanistically important mediator underlying the high prevalence of severe anemia among patients with HIV-associated tuberculosis, especially those with disseminated disease.

## Supplementary Data

Supplementary materials are available at http://jid.oxfordjournals.org. Consisting of data provided by the author to benefit the reader, the posted materials are not copyedited and are the sole responsibility of the author, so questions or comments should be addressed to the author.

Supplementary Data
