# Supplementary material for: Relationship Between Blood Concentrations of Hepcidin and Anemia Severity, Mycobacterial Burden, and Mortality Among Patients With HIV-Associated Tuberculosis
Source: J Infect Dis. 2015 Jul 1;213(1):61–70. doi: 10.1093/infdis/jiv364 (PMC4676545; doi:10.1093/infdis/jiv364)
Supplement: Supplementary Data [file supp_213_1_61__index.html]

Relationship Between Blood Concentrations of Hepcidin and Anemia Severity, Mycobacterial Burden, and Mortality Among Patients With HIV-Associated Tuberculosis — Supplementary Data 

# Relationship Between Blood Concentrations of Hepcidin and Anemia Severity, Mycobacterial Burden, and Mortality Among Patients With HIV-Associated Tuberculosis

## Supplementary Data

Supplementary Data

- Supplementary Data - Docx file
