# Supplementary material for: Relationship Between Blood Concentrations of Hepcidin and Anemia Severity, Mycobacterial Burden, and Mortality Among Patients With HIV-Associated Tuberculosis
Source: J Infect Dis. 2015 Jul 1;213(1):61–70. doi: 10.1093/infdis/jiv364 (PMC4676545; doi:10.1093/infdis/jiv364)
Supplement: Supplementary Data [file supp_jiv364_jiv364supp.docx]

**Supplementary Table 1.**  Relationship between hepcidin concentrations and TB assay results from different anatomical sites among TB+HIV+Ambulatory patients

|  | **Hepcidin**  **Median (IQR)** | **Fold-difference**  **(P-value)** |
| --- | --- | --- |
| **Respiratory samples** |  |  |
| **Sputum smear (n=58)** |  |  |
| Positive (n=18) | 23.0 (15.2-55.6) | **1.5**  (0.085) |
| Negative (n=40) | 15.6 (7.8-42.0) |  |
| **Sputum Xpert (n=55)** |  |  |
| Positive (n=38) | 28.5 (12.1-72.8) | **2.8**  (<0.001) |
| Negative (n=17) | 10.2 (6.7-12.2) |  |
| **Urine Xpert (n=49)** |  |  |
| Positive (n=12) | 54.6 (36.9-99.4) | **4.6**  (<0.001) |
| Negative (n=37) | 11.8 (8.3-21.7) |  |
